# Optimizing Two-Color Semiconductor Nanocrystal Immunoassays in Single Well Microtiter Plate Formats

**DOI:** 10.3390/s110807879

**Published:** 2011-08-11

**Authors:** Kim E. Sapsford, Samantha Spindel, Travis Jennings, Guoliang Tao, Robert C. Triulzi, W. Russ Algar, Igor L. Medintz

**Affiliations:** 1 Division of Biology, Office of Science and Engineering Laboratories, U.S. Food and Drug Administration, 10903 New Hampshire Avenue, Silver Spring, MD 20993, USA; E-Mail: samantha.spindel@fda.hhs.gov; 2 Fischell Department of Bioengineering, University of Maryland, College Park, MD 20993, USA; 3 eBioscience, Inc., 10255 Science Center Drive, San Diego, CA 92121, USA; E-Mails: travis.jennings@ebioscience.com (T.J.); guoliang.tao@ebioscience.com (G.T.); robert.triulzi@ebioscience.com (R.C.T.); 4 Center for Bio/Molecular Science and Engineering, Code 6900, U.S. Naval Research Laboratory, 4555 Overlook Avenue, S.W., Washington, DC 20375, USA; E-Mail: russ.algar.ctr.ca@nrl.navy.mil; 5 College of Science, George Mason University, 4400 University Dr., Fairfax, VA 22030, USA

**Keywords:** quantum dot (QD), nanocrystal (NC), semiconductor, bioconjugation, sensor, multiplex, immunoassay, sulfhydryl chemistry

## Abstract

The simultaneous detection of two analytes, chicken IgY (IgG) and Staphylococcal enterotoxin B (SEB), in the single well of a 96-well plate is demonstrated using luminescent semiconductor quantum dot nanocrystal (NC) tracers. The NC-labeled antibodies were prepared via sulfhydryl-reactive chemistry using a facile protocol that took <3 h. Dose response curves for each target were evaluated in a single immunoassay format and compared to Cy5, a fluorophore commonly used in fluorescent immunoassays, and found to be equivalent. Immunoassays were then performed in a duplex format, demonstrating multiplex detection in a single well with limits of detection equivalent to the single assay format: 9.8 ng/mL chicken IgG and 7.8 ng/mL SEB.

## 1. Introduction

Fluorescently labeled antibodies represent key reagents for numerous bioanalyses, including immunoassays and immunostaining, and are used in a myriad of applications that range from medical diagnosis through biological warfare agent monitoring. Despite progress in the development of bioanalyses based on synthetic biorecognition elements such as nucleic acid aptamers [1] and molecularly imprinted polymers [2], antibodies continue to offer the highest levels of affinity and specificity. Increasingly, there has been a push towards multiplexed analysis (*i.e.*, the simultaneous detection of multiple targets) to increase throughput, reduce cost and time, increase data generation, provide a better fundamental understanding, and simplify assay formats. However, one of the major impediments to multiplex immuno-based analysis has been the photophysical properties of the organic dyes most commonly used as fluorescent labels [3,4]. Limitations often arise from emission profile overlap between different dyes, the need for multiple excitation sources/wavelengths, and self-quenching at higher degrees of protein labeling. Semiconductor quantum dot nanocrystals (referred to herein as NC) are increasingly being incorporated into multiplex bioanalyses to address these shortcomings, wherein researchers take advantage of the broad absorption and sharp emission profiles of NCs, as well as their large extinction coefficients, excellent quantum yields and good photostability [5–7]. The broad NC absorption profile allows the use of a single excitation source to simultaneously excite NCs of different colors, and thus greatly simplifies the optical detection platform required for multiplex measurements. Moreover, the sharp photoluminescence (PL) emission allows those different colors to be resolved with minimal crosstalk or, when many colors are used, straightforward Gaussian deconvolution algorithms. As such, NCs have demonstrated great promise in multiplex immunoassays and immuno-based staining of western blots or tissue samples [7–15].

The principal impediment to the routine use of NCs as fluorophores in immuno-based bioanalyses continues to be the limited chemistries that are currently available for attaching biologicals, such as antibodies and proteins, to the NC surface. The chemistry should be facile and well controlled in order to reproducibly yield functional NC-bioconjugates. Typical chemistries target the ubiquitous amine and carboxyl groups of proteins, and occasionally the less prevalent sulfhydryl groups that can be present naturally or introduced through recombinant and chemical methods [16–18]. To date, the most common chemistries used to prepare NC-bioconjugates for multiplex applications include: (1) carboxylated NCs activated via carbodiimide (EDC)/succinimidyl ester (NHS) chemistry, which targets the amine groups of antibodies or protein A [9,12]; (2) biotin-labeled NCs that bind streptavidin-modified species [15]; and (3) amine modified NCs that are coupled with protein amine groups via glutaraldehyde [11]. These chemistries typically require extensive optimization and are plagued with several issues—most significantly, susceptibility to undesirable cross-linking that often results in heterogeneous conjugate architecture, aggregation, and mixed avidity of the final product. Targeting the less prevalent sulfhydryl groups or, alternatively, incorporating unique chemical or affinity handles, can alleviate some of these issues [10,19–21]. For example, Zahavy and coworkers developed a multi-step protocol for modifying amine modified NCs with antibodies. The NCs were activated with the heterobifunctional crosslinker 4-(maleimidomethyl)-1-cycohexanecarboxylic acid N-hydroxysuccinimide ester (SMCC), converting the amines to sulfhydryl-reactive maleimides [10]. Antibodies were pre-reduced with dithiothreitol (DTT) to yield free sulfhydryl groups at the hinge region, exposed to the activated NCs, and the conjugates purified by size exclusion chromatography. The resulting NC-labeled antibodies were used in a multiplex flow cytometry analysis of pathogenic bacteria. Alternative strategies for controlled bioconjugation include polyhistidine-based self-assembly, arsenic-vicinal tetracysteine motifs, or ‘click’ chemistry. These tend to require extensive protein and/or NC engineering, and are currently much less utilized. Therefore, it remains highly desirable to develop novel chemistries that target the amine and sulfhydryl functionalities of biomolecules while simultaneously offering improved control over bioconjugate architecture [8,19–21].

**Figure 1.**
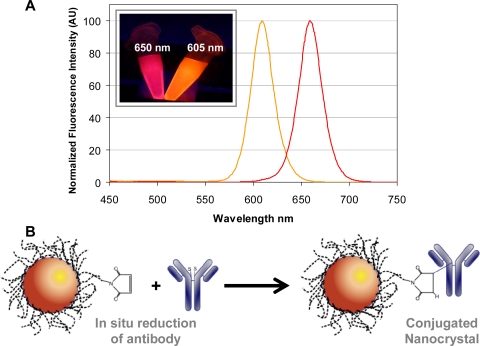
Sulfhydryl-reactive conjugation chemistry. **(A)** Photoluminescence spectra for eFluor® NC605 and NC650 used in this study, Ex@400 nm, the insert shows a digital photographic image of NCs in solution under UV 365 nm excitation. **(B)** Schematic of the sulfhydryl-reactive chemistry, which consists of a maleimide-functionalized NC and an *in situ* reducing agent. Antibodies containing disulfide bonds are directly reduced in solution with reactive NCs for immediate conjugation. Note: not to scale.

In this work, we introduce a sulfhydryl-reactive NC-bioconjugation chemistry that overcomes many of the issues mentioned above, see Figure 1. The chemistry, outlined in Figure 1(B), is a one-step reaction that utilizes proprietary maleimide-activated NCs and an *in situ* antibody reduction method developed by eBioscience. While the protocol developed by Zahavy and coworkers also targeted sulfhydryl groups, it was more complex, requiring multiple activation and purification steps [10]. The chemistry used herein was rapidly implemented and yielded purified NC-antibody conjugates in less than 3 h. The conjugates had good retention of binding activity and little-to-no aggregation was observed. The utility of the NC-antibody conjugates was demonstrated in single and duplex immunoassay formats that used two different colors of eBioscience NC. As illustrated in Figure 1(A), the emission maxima of the NCs were centered at 605 ± 3 nm (NC605) and 650 ± 3 nm (NC650), and provided spectrally resolved multicolor detection with minimal crosstalk. For this initial proof-of-concept study, SEB and chicken IgG immunoassays were selected as targets since they have been shown in previous fluorescent immunoassay studies and control experiments (data not shown) to be highly selective with no observable cross-reactivity, thus negating this potentially complicating issue [7,8–10]. It is also important to note that SEB is a protein toxin, generated by *Staphylococcus aureus* bacterium, and its generalized detection remains of high interest due to its common association with food poisoning. Rabbit anti-chicken IgG antibodies were labeled with NC605 and rabbit anti-SEB antibodies with NC650 for use as tracers. The dose response and limit of detection (LOD) for the NC-based single immunoassays compared favorably with those obtained using a standard organic fluorophore, Cy5. Importantly, the NC-based single sandwich immunoassays were readily adapted to a multiplex format for the simultaneous detection of two target antigens.

## 2. Experimental Section

### 2.1. Materials

Staphylococcal enterotoxin B (SEB) and affinity purified rabbit anti-SEB were purchased from Toxin Technology Inc. (Sarasota, FL, USA). Rabbit anti-chicken IgG (IgY) and Chicken IgG were purchased from Jackson ImmunoResearch Laboratories Inc (West Grove, PA, USA). Phosphate buffered saline (PBS), Corning Costar® flat bottom high binding white 96-well assay plates, Thermal Seal® sealing film for 96-well plates, dimethyl sulfoxide (DMSO) and bovine serum albumin (BSA) were obtained from Sigma-Aldrich (St. Louis, MO, USA). Millipore Amicon® Ultra centrifugal filter devices 100 kDa were purchased from Millipore Corporation (Billerica, MA, USA). The eFluor® nanocrystals (NC) NC605-maleimde and NC650-maleimde were prepared and supplied by eBioscience (San Diego, CA, USA). Amersham™ Cy™5 mono-reactive dye was purchased from GE Healthcare Bio-Sciences Corp. (Piscataway, NJ, USA). Zebra™ desalt spin columns (2 mL) were obtained from Pierce Biotechnology Inc., part of Thermo Fisher Scientific Inc (Rockford, IL, USA). Doubly distilled water (ddH_2_O) was used throughout the experiments and was prepared in-house using a Nanopure Diamond™ water purification system (Barnstead, Dubuque, IA, USA).

### 2.2. Nanocrystals

The eBioscience eFluor® CdSe/ZnS core/shell NCs with emission maxima centered at 605 nm (NC605) and 650 nm (NC650) were synthesized using standard high temperature reactions of organometallic precursors in hot coordinating solvents [21,22]. NCs were made water soluble using a DSPE-PEG lipid (1,2-distearoyl-sn-glycero-3-phosphoethanolamine-*N*-[carboxy(polyethylene glycol)-2000]) as previously described [23,24]. NC surfaces were further modified with a reactive-maleimide using proprietary techniques at eBioscience (San Diego, CA, USA). After maleimido-activation, the NCs were lyophilized to dryness and stored under vacuum at 4 °C until use. NCs prepared in this manner remain stable for at least 6–12 months.

### 2.3. Labeling Antibodies with Cy5 Dye

Antibodies were labeled with Cy5 mono-reactive dye via the primary amines on the protein. First, the rabbit (Rb) anti-SEB and rabbit anti-chicken IgG were prepared at 0.5 mg/mL (total 600 μL) in PBS and one vial of the Cy5 mono-reactive dye resuspended in 50 μL DMSO. Cy5 (7.5 μL) was then added to each of the antibody solutions and allowed to react at room temperature (RT) for ∼30 min. The unincorporated dye was removed from the Cy5-labeled antibody using a Zebra™ desalt spin column following the instructions provided by the manufacturer. The final antibody concentration and dye-to-antibody ratio was determined by measuring the absorbance of the purified Cy5-labeled antibody at 280 and 650 nm using an Amersham Biosciences Ultrospec 2100 pro UV/visible Spectrophotometer (GE Healthcare, Piscataway, NJ, USA).

### 2.4. Labeling Antibodies with eBioscience NCs

Antibodies were labeled with the eBiosceinces eFluor® NCs using the sulfhydryl-reactive conjugation reagents and instructions provided by the manufacturer. Briefly, lyophilized NCs were reconstituted in 200 μL Conjugation Buffer by heating the mixture on high in a microwave for 5–10 s, repeating 3–4 times as necessary. A total of 200 μg of antibody was then added to the NCs and the reaction incubated for 2 h at RT on a shaker. The antibody:NC ratios during reaction were 2:1 for the NC605 and 4:1 for the NC650. For the immunoassay duplex studies, NC605-maleimide and NC650-maleimide NCs were conjugated to rabbit anti-chicken IgG and rabbit anti-SEB, respectively. After 2 h, the reaction was stopped by adding 0.7 μL of Quencher (2-mercaptoethanol) directly to the mixture, vortexing and incubating for an additional 10 min on the shaker. The resulting antibody-conjugated NCs were purified using a 100 kDa centrifugal filter unit. The reaction mixture was added to a centrifugal filter unit (pre-equilibrated with Purification Buffer), the volume increased to a total of 1 mL using Purification Buffer, then spun at 1,000 g for 10 min (Beckman CS-6KR Centrifuge). Once the sample volume had reduced to ∼0.1 mL, an additional 1 mL of Purification Buffer was added and the spin repeated, and this process was repeated for a total of four spins. The NC-antibody conjugate was then transferred to a 1.5 mL microcentrifuge tube and spun at 3,000 g for 5 min to remove any aggregates. The purified NC-antibody conjugate was characterized using UV-visible spectroscopy and stored at 4 °C prior to use. Final NC concentration refers to that determined from the QD. Where indicated, subsequent dilutions are of this concentration.

### 2.5. Patterning 96-Well Plates with Capture Antibodies

High binding white 96-well assay plates were functionalized with capture antibodies prior to immunoassays. Wells were functionalized with either; (1) a single capture antibody species, by adding 50 μL per well of 10 μg/mL rabbit anti-chicken IgG or rabbit anti-SEB in PBS, or (2) multiple capture species, by adding 50 μL of a capture antibody mix in PBS. The capture antibody mix comprised 7.5 μg/mL rabbit anti-chicken IgG and 2.5 μg/mL rabbit anti-SEB in PBS. The wells were sealed with Thermal Seal® sealing film and incubated for ∼1 h at RT and then overnight at 4 °C. The wells were then emptied, washed four times with 200 μL/well ddH_2_O, then filled with 200 μL/well PBS + 1% BSA and blocked at RT for ∼1.5 h on a rocker (The Belly Dancer, Stovall LifeScience, Inc, Greensboro, NC, USA).

### 2.6. Immunoassays

Following blocking, the solution was removed from the wells and 50 μL/well of the appropriate sample was added. Depending on the capture antibodies in the microtiter plate, samples for the single immunoassays comprised either chicken IgG (C: 0–2.5 μg/mL) or SEB (S: 0–0.5 μg/mL) in PBS + 0.1% BSA. These were left to incubate on a rocker for 1.5 h at RT. The wells were then washed with PBS (4 × 200 μL/well) before the tracer was added at 50 μL/well and incubated for 1 h at RT on the shaker. The single immunoassay tracers consisted of either NC605-rabbit anti-chicken IgG (1:400 dilution), Cy5-rabbit anti-chicken IgG (10 μg/mL), NC650-rabbit anti-SEB (1:100 dilution), or Cy5-rabbit anti-SEB (10 μg/mL) in PBS + 0.1% BSA. The wells were then washed with 2 × 200 μL/well PBS followed by 2 × 200 μL/well ddH_2_O and dried with air. Samples for the duplex immunoassay comprised mixed concentrations of chicken IgG (C: 0−2.5 μg/mL) and SEB (S: 0−0.5 μg/mL) in PBS + 0.1% BSA. These were left to incubate on a rocker for 1.5 h at RT. The wells were then washed with PBS (4 × 200 μL/well) before the tracer mix was added at 50 μL/well and incubated for 1 h at RT on the shaker. The duplex immunoassay tracer mix consisted of NC605-rabbit anti-chicken IgG (1:400 dilution) and NC650-rabbit anti-SEB (1:100 dilution) NCs in PBS + 0.1% BSA. The wells were then washed with 2 × 200 μL/well PBS followed by 2 × 200 μL/well ddH_2_O and dried with air. The fluorescence intensity and fluorescence intensity wavelength scans were recorded using a Tecan Infinite M1000 Dual Monochromator Multifunction Plate Reader (Tecan, Research Triangle Park, NC, USA) using an excitation of 400 nm for the NC-labeled tracer exposed wells and 650 nm for wells containing Cy5-labeled tracers.

## 3. Results and Discussion

### 3.1. Single Immunoassays

**Figure 2.**
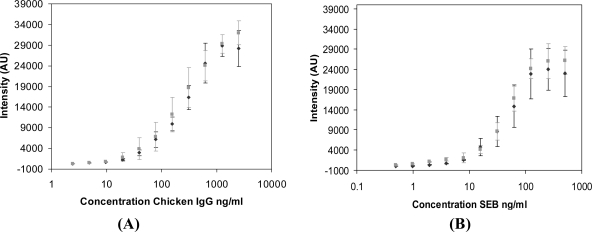
Single format NC tracers versus Cy5 tracer. (A) Chicken immunoassay, microtiter well plate functionalized with single species capture antibodies for chicken IgG; the tracer was either 10 μg/mL Cy5 rabbit-anti-Chicken IgG (♦) or NC605-rabbit-anti-Chicken IgG (▪) at 1:400 dilution. **(B)** SEB immunoassay, microtiter well functionalized with single species capture antibodies for SEB; the tracer was either 10 μg/mL Cy5 rabbit-anti-SEB (♦) or NC650-rabbit-anti-SEB (▪) at 1:100 dilution. Intensity measurements were recorded at the emission maxima (Em) for each of the particular fluorescent labels: NC605 Ex@400 nm, Em@605 nm; NC650 Ex@400 nm, Em@650 nm; Cy5 Ex@650 nm, Em @670 nm (n ≥ 4, for all data).

Prior to investigating the multiplex capabilities of the NC materials, assay conditions were optimized using a single sandwich immunoassay format. The analytical figures of merit obtained with the NC-labeled antibodies were compared to antibodies labeled with Cy5. High-binding microtiter (96-well) plates were first functionalized with capture antibodies for either chicken IgG or SEB, then exposed to the target antigen and subsequently incubated with either Cy5- or NC-labeled tracer antibodies. Following extensive washing, the plates were dried, and fluorescence intensities were measured at the emission maxima of the specific fluorescent label used (*i.e.*, NC605 at 605 nm; NC650 at 650 nm; Cy5 at 670 nm). While the Cy5-labeled antibodies were used at 10 μg/mL, a range of NC-antibody conjugate dilutions were initially investigated (from 1:50–1:600) to determine the optimal dilution for each target immunoassay (data not shown). Figure 2 shows the resulting dose response curves for chicken IgG (A) and SEB (B), comparing the Cy5- and the optimized NC-labeled tracer antibodies for each analyte. Clearly, there is good agreement between the Cy5- and NC-tracers for both the chicken IgG and SEB immunoassays. The data were fit with a four-parameter logistic model commonly applied to immunoassay dose response curves, and a statistical comparison of the fit parameters was done. At the 99% confidence level (t-test, *p* > 0.01), the fit parameters that determine the EC_50_ value and slope of response for each immunoassay (chicken IgG and SEB) were not significantly different between the use of Cy5 or NCs. The limit of detection (LOD) determined for each immunoassay is listed in Table 1. The LOD was defined as the antigen concentration that generated a signal greater than three standard deviations above of the background signal. While the LOD obtained using a Cy5 tracer (4.9 ng/mL) in the chicken IgG immunoassay was slightly better than that obtained with the NC605 (9.8 ng/mL), an LOD of 7.8 ng/mL was obtained in the SEB immunoassay using both the Cy5 and NC650 tracers. Overall, the similarity of the fit parameters and the LOD between the use of Cy5 and NCs are good indicators of controlled NC-antibody conjugate preparation, and retention of binding activity.

### 3.2. Duplex Immunoassays

**Figure 3.**
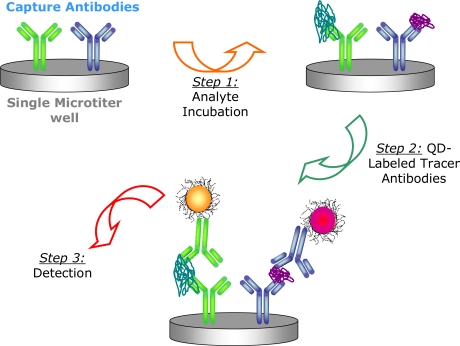
Scheme illustrating duplex detection in a single well of a microtiter plate using NC-based tracers. Not to scale.

Given the success of the NCs in single immunoassays, a duplex immunoassay was subsequently performed to evaluate the performance of NC-antibody tracer probes under multiplex conditions. The assay is illustrated in Figure 3 and utilizes the co-immobilization of the anti-chicken IgG and anti-SEB. Microtiter plate wells were functionalized with capture antibodies at anti-chicken IgG: anti-SEB ratios of 7.5:2.5 and 9.0:1.0 μg/mL. The ratio 7.5:2.5 was found to be optimal (data not shown). Once the capture antibodies were immobilized, the wells were blocked and exposed to different concentrations of both chicken IgG and SEB analyte. In the target samples, chicken IgG was increased from 0 to 2,500 ng/mL while SEB was simultaneously decreased from 500 to 0 ng/mL. Detection was done in parallel using the corresponding NC605 and NC650 tracer antibody conjugates. The wells were washed and incubated for 1 h with a mixed solution of the NC tracers at the optimized dilutions determined from the single immunoassay studies.

**Figure 4.**
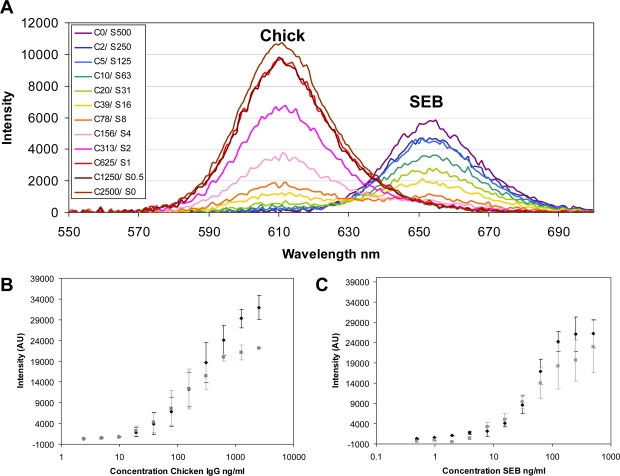
Duplex immunoassays. **(A)** Chicken and SEB immunoassay, microtiter well functionalized with capture antibodies for both chicken IgG (7.5 ug/mL) and SEB (2.5 ug/mL); the tracer comprised NC605-Rb-anti-Chicken IgG (1:400 dilution) and NC650-Rb-anti-SEB (1:100 dilution). The chicken (C) and SEB (S) concentrations exposed are indicated in ng/mL. **(B)** Dose response for the Chicken immunoassay in a single format versus duplex format. Single capture species combined with single NC-tracer (♦). Duplex capture species combined with duplex NC-tracers ( 
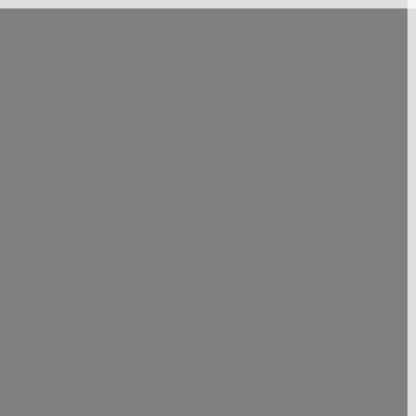
). **(C)** Dose response for the SEB immunoassay in a single format versus duplex format. Single capture species combined with single NC-tracer (♦). Duplex capture species combined with duplex NC-tracers ( 
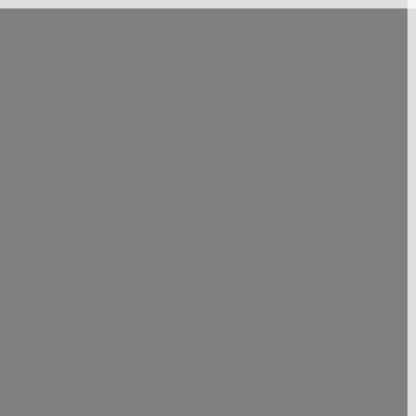
). Intensity measurements were recorded at the emission maxima (Em) for each of the particular fluorescent labels: NC605 Ex@400 nm, Em@605 nm; NC650 Ex@400 nm, Em@650 nm (n ≥ 4, for all data).

Figure 4 shows representative data obtained from fluorescence measurements after final washing of the wells. The excellent spectral separation of the NC605 and NC650 emissions allowed resolution of the relative changes in target antigen concentrations across the entire range of samples (Figure 4(A)). An important advantage of the method was that the duplex data was collected in parallel using only 400 nm as an excitation wavelength. If the assay had been done using, for example, Cy3 and Cy5 tracers, both green and red excitation wavelengths would have been necessary. This would necessitate the integration of two parallel excitation sources (e.g., lasers) into the fluorescence instrumentation or, alternatively, serial acquisition of the two different fluorescence signals. Figure 4(B,C) shows intensity data versus concentration for the chicken IgG and SEB antigens, respectively, along with a side-by-side comparison to the corresponding data from the single NC tracer immunoassay formats. The duplex LOD (Table 1) were 9.8 ng/mL chicken IgG and 7.8 ng/mL SEB. These values are equivalent to the LOD obtained using the single immunoassay format and clearly demonstrate that switching to a duplex format was not detrimental to the assay sensitivity. Due the broad interest in detection as a food toxin, many immunoassay-based methods for the detection of SEB have been developed with reported LODs ranging from, 4 ng/mL down to <2.5 fg/mL depending upon the exact assay protocol implemented [25–31]. While the LOD for SEB observed here is not as sensitive as some of these reported values, this was not our principal goal. Rather, our focus was on proof of concept for this two-color NC labeling and detection scheme. We anticipate that further improvements to this assay format along with selection of higher-affinity antibodies can help to improve the sensitivity quite significantly and this is currently under investigation in both single and multiplexed formats.

Given that the two different colors of NC were spectrally separated by approximately 50 nm, it is expected that a pentaplex immunoassay will be possible using NC tracers within the spectral range of 450–650 nm, while still providing a large effective Stokes’ shift that minimizes background. The use of NCs as labels should provide similar advantages in other bioanalysis formats that use antibody tracer agents, such as flow cytometry and immunohistochemistry.

## 4. Conclusions

NCs have several unique properties that make them ideal as fluorescent labels for a variety of applications. In particular, their broad absorption and narrow emission profiles are excellently suited for multiplexing. Current methods of labeling these NC materials are often complex and prone to aggregation and the loss of product. The facile labeling method used herein targeted antibody cysteine residues with maleimide active NCs using a protocol that took less than 3 h. Through the use of two different color NCs with emissions centered at 605 and 650 nm, we demonstrated the simultaneous immunoassay detection of two analytes, chicken IgY (IgG) and Staphylococcal enterotoxin B (SEB), in a single well of a 96-well plate. Dose response curves for each target were first evaluated in a single format using the NCs, and compared to Cy5, a fluorophore commonly used in fluorescent immunoassays. The resulting dose response curves were statistically equivalent between the two fluorescent labels at the 99% confidence level. The immunoassay was then preformed in a duplex format, demonstrating multiplexed detection in a single well with LOD equivalent to the NC single immunoassay format: 9.8 ng/mL chicken IgG and 7.8 ng/mL SEB. Furthermore, energy transfer (FRET) between both nanocrystals was not evident in the duplex system, as shown by the LOD equivalency between single and multiplexed formats. This strongly suggests that similar NC immunoassays may be amenable to much denser color or ‘deeper’ multiplexing (≥3) configurations, especially in areas of the spectral window not accessed here and indeed preliminary attempts at this are also underway [32]. The ability to bioconjugate not only NCs but almost all nanoparticulate materials in general in an intimate manner while allowing for careful control over all requisite parameters such as ratio, orientation, separation distance and function cannot be overstated as it is one of the long-term critical needs in bionanotechnology [32–34].

## Figures and Tables

**Table 1.** Limits of detection (LOD) determined from the dose response studies.

**Target**	**Tracer**	**Format**	**LOD (ng/mL)**	**n**

	Cy5	Single	4.9	8
Chicken IgG	NC605	Single	9.8	8
	NC605	Duplex	9.8	4

	Cy5	Single	7.8	4
SEB	NC650	Single	7.8	4
	NC650	Duplex	7.8	4
